# Wasserstein GAN-based estimation for conditional distribution function with current status data

**DOI:** 10.1007/s10985-026-09691-4

**Published:** 2026-01-31

**Authors:** Wen Su, Changyu Liu, Guosheng Yin, Jian Huang

**Affiliations:** 1 Department of Biostatistics, City University of Hong Kong, Hong Kong, China; 2 Department of Applied Mathematics, The Hong Kong Polytechnic University, Hong Kong, China; 3 Department of Statistics and Actuarial Science, The University of Hong Kong, Hong Kong, China

**Keywords:** Current status data, Generative learning, Neural networks, Nonparametric estimation, Wasserstein distance, 62G05

## Abstract

Current status data are commonly encountered in modern medicine, econometrics and social science. Its unique characteristics pose significant challenges to the analysis of such data and the existing methods often suffer grave consequences when the underlying model is misspecified. To address these difficulties, we propose a model-free two-stage generative approach for estimating the conditional cumulative distribution function given predictors. We first learn a conditional generator nonparametrically for the joint conditional distribution of observation times and event status, and then construct the nonparametric maximum likelihood estimators of conditional distribution functions based on samples from the conditional generator. Subsequently, we study the convergence properties of the proposed estimator and establish its consistency. Simulation studies under various settings show the superior performance of the deep conditional generative approach over the classical modeling approaches and an application to Parvovirus B19 seroprevalence data yields reasonable predictions.

## Introduction

Interval censored data often arise in medical, public health, economic studies, and social sciences. Due to interval censoring, the failure time cannot be observed exactly but is observed only to belong to an interval. If either the left boundary of the interval is zero or the right boundary is infinity, it is known as current status data or case I interval-censored data (Groeneboom and Wellner 1992; Sun 2006). That is, the failure time variable of interest is observed only once, and it is known only to be either smaller or greater than the observation time. For example, a large seroprevalence survey investigated the presence of Parvovirus B19 virus in serum samples provided by five national centers in Belgium, Finland, England & Wales, Italy, and Poland between 1995 and 2004 (Mossong et al. 2008). The event of interest is the immunological status of the sera and for seropositive samples, the exact timing of contracting the virus is not available. Thus the analysis of such data becomes extremely challenging due to its unique characteristics and requires dedicated tools. For a comprehensive review on interval-censored data analysis, refer to Sun (2006).

Many nonparametric and semiparametric methods have been developed to tackle the difficulties arising from current status data. Groeneboom and Wellner (1992) and Huang and Wellner (1995) derived the limiting distribution of nonparametric maximum likelihood estimator (NPMLE) for current status data. Banerjee and Wellner (2001) developed a likelihood ratio test to make inference for an unknown distribution function. Groeneboom et al. (2010) and Groeneboom (2014) proposed the maximum smoothed likelihood estimator; Groeneboom and Jongbloed (2014, 2015) further discussed these nonparametric estimators and the likelihood ratio test. Groeneboom and Hendrickx (2018) and Kim et al. (2022) constructed confidence intervals for a cumulative distribution function with current status data. To address current status data exhibiting various characteristics, Ma (2009) investigated the cure rate model; Tang et al. (2012) developed an NPMLE for grid observation times with ties; Liang et al. (2017) analyzed repeated current status data; Li et al. (2019) introduced estimation of the additive hazards model while accounting for informative censoring. Additionally, Ou et al. (2014) studied quantile regression models for current status data; Lu et al. (2016) developed the partially linear single-index proportional hazards model. More recently, Lu et al. (2020) introduced a penalized approach for a semiparametric linear transformation model; Lam et al. (2020) investigated current status data with informative cluster size using the generalized estimation equation approach; Wang et al. (2020) investigated current status data models through latent variables; Travis-Lumer and Goldberg (2021) implemented the kernel machine approach to estimating failure time expectation; Yang et al. (2021) developed survival trees based on the classification and regression tree (CART) algorithms; Chen et al. (2021) focused on eliminating bias caused by a misclassified covariate using semiparametric AFT models. Rapid growth in computational power and developments of machine learning tools opened up a myriad of new possibilities for the analysis of current status data, which enable us to build sumptuous extensions on traditional approaches. Since the introduction of generative adversarial network (GAN) (Goodfellow et al. 2014), many extensions such as conditional GAN (cGAN) (Mirza and Osindero 2014) and Wasserstein GAN (WGAN) (Arjovsky et al. 2017) have been developed. Gui et al. (2015) provided an overview of algorithms, theory, and applications on GAN. Recently, Zhou et al. (2021) proposed a conditional generator estimated with deep neural networks using the Kullback–Liebler divergence to match appropriate joint distributions, while Liu et al. (2021) considered Wasserstein distance as a measure of discrepancy between the conditional distribution and target joint distribution in a generative learning approach. Lately, applications of GAN to survival data have been gaining much attention. For instance, Chapfuwa et al. (2018) applied deep adversarial learning tools to address nonparametric estimation for time-to-event distributions; Zhong et al. (2021) developed tools for deep survival analysis and established the consistency and convergence rate of the proposed survival function estimator. Furthermore, Zhong et al. (2022) studied the partially linear Cox model using neural network and established the asymptotic properties of the maximum partial likelihood estimator; Zhou et al. (2022) introduced a two-step generative approach to analyzing right-censored survival data, which first learns a conditional generator nonparametrically, and then obtains the Kaplan–Meier and Nelson–Aalen estimators.

Inspired by recent developments in GAN (Goodfellow et al. 2014) and WGAN (Arjovsky et al. 2017), we propose a novel generative approach for analyzing current status data. While traditional methods often lead to inaccurate results when the underlying model is misspecified, we introduce a model-free generative conditional distribution estimator (GCDE) that consists of two steps. First, a conditional generator is estimated based on the joint conditional distribution of observation times and censoring indicator given covariates, then a maximum likelihood estimator (MLE) of the cumulative distribution function is constructed using samples generated from this conditional generator. Hence a highlight of our proposed method is its capability of making predictions on censoring status of subjects. To the best of our knowledge, no existing methods can generate predictions on censoring rates for current status data. Application to the Parvovirus B19 study data in Sect. 6 shows that GCDE generated reasonable distribution functions and censoring indicators with high accuracy for subjects in the testing set.

The remainder of this paper is organized as follows. We present the GCDE methodology including a detailed description of the two-step estimation procedure and provide implementation technicalities in Sect. 2. We establish the asymptotic consistency in Sect. 3. In Sect. 4, we report results from simulation studies with comparisons to the Cox proportional hazards model and the AFT model under various settings. Section 5 shows application of the proposed method to the Parvovirus B19 seroprevalence survey data. Some concluding remarks are included in Sect. 6 and details of proofs are relegated to the Appendix.

## WGAN-based estimation

Let $T\in \mathbb {R}^+$ be a failure time such as the (unobserved) time of onset of a disease, $Y \in \mathbb {R}^+$ be an examination time, and $X \in \mathcal{X}\subseteq \mathbb {R}^d$ be a *d*-dimensional predictor. At the examination times, we cannot observe *T* directly, but only observe $\Delta =1\{T\le Y\}$. Together with the predictor *X*, the observed data are $(Y, \Delta , X)$ for an individual under study. The failure time *T* and the observation time *Y* are conditionally independent given the covariate *X*. For given $X=x$, let $F^x$ denote the conditional distribution of the failure time *T*, i.e., $F^x(t)=P(T\le t|X=x)$. We propose a two-stage GAN-based approach to estimating $F^x$ nonparametrically.

### Stage 1: Estimation of the conditional generator

Let $P_{(Y, \Delta )\mid X}$ denote the joint conditional distribution of $(Y, \Delta )$ given *X*. The first stage of the proposed approch is to learn a conditional generator for the joint conditional distribution $P_{(Y, \Delta )\mid X}$ of the examination time *Y* and the indicator $\Delta$ given covariate *X*.

By the noise outsourcing lemma (Theorem 5.10 in Kallenberg (2002)), there exists a function $G=(G_1, G_2): \mathbb {R}^s \times \mathbb {R}^d \rightarrow \mathbb {R}^+ \times \{0,1\}$ and a random variable $\eta \sim P_{\eta }$ in $\mathbb {R}^s$ independent of *X* such that

1 $$\begin{aligned} (X, G_1(\eta , X), G_2(\eta , X)) = (X, Y, \Delta ) \ \text { almost surely}. \end{aligned}$$

Denote the joint distribution of $(X, G_1(\eta , X), G_2(\eta , X))$ by $P_{X,G_1, G_2}.$ Since $\eta$ and *X* are independent, $(G_1(\eta , x), G_2(\eta , x)) \sim P_{(Y, \Delta )\mid X=x}$ if and only if (1) holds. Therefore, to search the conditional generator, it is equivalent to find $(G_1^*, G_2^*)$ such that the joint distribution $P_{X,G_1^*, G_2^*}(X, G_1^*(\eta , X), G_2^*(\eta , X))$ matches the joint distribution $P_{X, Y, \Delta }(X, Y, \Delta )$.

To achieve this goal, we need a suitable divergence measure between the distributions $P_{X, G_1, G_2}$ and $P_{X, Y, \Delta }.$ Many different divergence measures for probability distributions have been developed. A commonly used measure is the *f*-divergence (Ali and Silvey 1966), which includes the Kullback–Liebler and Jensen–Shannon divergences as special cases. Recently, the Wasserstein distance has attracted great attention. For example, the 1-Wasserstein distance was used in the context of generative learning (Arjovsky et al. 2017). The Wasserstein distance can metricize the space of probability distributions under mild conditions, while other discrepancy measures, including the Kullback–Liebler and Jensen–Shannon divergences, do not have such features. In particular, the computation of the Wasserstein distance does not involve any density function, so we choose a divergence measure to be the 1-Wasserstein distance to learn distributions without density functions.

For two probability measures $\mu$ and $\nu$ with the finite first moment, the 1-Wasserstein distance is defined as

$$\begin{aligned} d_{W_1}(\mu , \nu )= \inf _{\gamma \in \Gamma (\mu , \nu )} \int \Vert u-v\Vert _1d\gamma (u, v), \end{aligned}$$

where $\Gamma (\mu , \nu )$ is the set of joint probability distributions with marginals $\mu$ and $\nu .$ Let $\mathcal {F}^1_{\text {Lip}}$ be the 1-Lipschitz class:

$$\begin{aligned} \mathcal {F}^1_{\text {Lip}}=\{f: \mathbb {R}^k \rightarrow \mathbb {R}, |f(u)-f(v)| \le \Vert u-v\Vert _2,\ u, v \in \mathbb {R}^k\}. \end{aligned}$$

The 1-Wasserstein metric is equivalent to the Monge–Rubinstein dual,

$$\begin{aligned} d_{W_1}(\mu , \nu )=\sup _{f \in \mathcal {F}^1_{\text {Lip}}} \left\{ \mathbb {E}_{U \sim \mu } f(U) - \mathbb {E}_{V\sim \nu } f(V) \right\} , \end{aligned}$$

which has a computationally convenient form (Villani 2008). The 1-Wasserstein distance between $P_{X, G_1, G_2}$ and $P_{X, Y, \Delta }$ is

$$\begin{aligned} d_{W_1}(P_{X, G_1, G_2}, P_{X, Y, \Delta }) = \sup _{D\in \mathcal {F}^1_{\text {Lip}}}\left\{ \mathbb {E}_{(X, \eta )} D(X,G_1(\eta ,X), G_2(\eta , X)) -\mathbb {E}_{(X, Y,\Delta )} D(X,Y, \Delta )\right\} . \end{aligned}$$

Clearly, we have $d_{W_1} (P_{X, G_1, G_2}, P_{X, Y, \Delta }) \ge 0$ for every measurable $G=(G_1, G_2)$ and the equality holds if and only if $P_{X, G_1, G_2}=P_{X, Y, \Delta }.$ Thus a sufficient and necessary condition for $(G_1^*, G_2^*) \in \mathop {\textrm{argmin}}\limits _{G_1, G_2} d_{W_1}(P_{X, G_1, G_2}, P_{X, Y, \Delta })$ is $P_{X, G_1^*, G_2^*} = P_{X, Y, \Delta }$, which implies $(G_1^*(\eta , x), G_2^*(\eta , x)) \sim P_{(Y, \Delta )\mid X=x},$ for any $x \in \mathcal {X}.$ Let $\mathcal {G}$ be the set of the feedforward generator networks, for $(G_1, G_2) \in \mathcal {G}$ and $D\in \mathcal {F}^1_{\text {Lip}}$, define

$$\begin{aligned} \mathcal {L}(G, D)=\mathbb {E}D(X,G_1(\eta ,X), G_2(\eta , X))-\mathbb {E}D(X,Y, \Delta ). \end{aligned}$$

Therefore, at the population level, we can transfer the problem of finding the conditional generator to a minimax problem:

$$\arg \min _{(G_1, G_2) \in \mathcal {G}} \max _{D \in \mathcal {F}^1_{\text {Lip}}} \mathcal {L}(G_1, G_2, D).$$

In a random sample of case I interval-censored observations, $\{(X_i, Y_i, \Delta _i), i=1, \ldots , n\}$ are i.i.d. copies of $(X, Y, \Delta ).$ Let $\{\eta _i, i=1,\ldots ,n\}$ be random variables independently generated from $P_{\eta }$. An empirical version of $\mathcal {L}(G, D)$ based on $(X_i, Y_i, \Delta _i)$ and $\eta _i, i=1,\ldots , n,$ is

$$\begin{aligned} \mathcal {L}_n(G,D) =&\frac{1}{n}\sum _{i=1}^n D(X_i, G_1(\eta _i,X_i), G_2(\eta _i, X_i)) - \frac{1}{n}\sum _{i=1}^n D(X_i,Y_i, \Delta _i) . \end{aligned}$$

Both the generator *G* and the discriminator *D* can be implemented by the feedforward ReLU neural networks, which can be represented in the form of $g=g_{L}\circ g_{L-1}\circ \cdots \circ g_{0},$ where $g_{i}(x)=\sigma (V_ix+b_i)$ and $g_{L}(x)=V_{L}x+b_{L}$, with the weight matrices $V_{i}\in \mathbb {R}^{d_{i+1}\times d_{i}}$ and the bias vectors $b_{i}\in \mathbb {R}^{d_{i+1}\times 1}$ for $i=0,\dots , L$, and $\sigma$ is the componentwise ReLU activation function. It follows that the network is parameterized by $(V_0,\dots , V_{L}, b_{0},\dots , b_{L})$ and has the depth (i.e., the number of hidden layers) equal to *L* and the width (the maximum width of all hidden layers) equal to $W=\max \{d_1,\dots , d_{L}\}$. Let $(L_1, W_1)$ be the depth and width of the feedforward ReLU generator network $G_{{\boldsymbol{\theta }}}=(G_{{\boldsymbol{\theta }}_1}, G_{{\boldsymbol{\theta }}_2})$ and let $(L_2,W_2)$ be those of the feedforward ReLU discriminator network $D_{{\boldsymbol{\phi }}}$. Denote the parameters of the conditional generator by ${\boldsymbol{\theta }}_1=(V_{1,0},\dots , V_{1,L_1},b_{1,0},\dots , b_{1,L_1})$ and ${\boldsymbol{\theta }}_2=(V_{2,0},\dots , V_{2,L_1},b_{2,0},\dots , b_{2,L_1})$. Denote the parameter of the discriminator by ${\boldsymbol{\phi }}=(V_{0},\dots , V_{L_2},b_{ 0},\dots , b_{L_2})$.

We use feedforward neural networks $(G_{{\boldsymbol{\theta }}_1}, G_{{\boldsymbol{\theta }}_2})$ with parameters $({\boldsymbol{\theta }}_1, {\boldsymbol{\theta }}_2)$ for estimating the conditional generator and another network $D_{{\boldsymbol{\phi }}}$ with parameter ${\boldsymbol{\phi }}$ for estimating the discriminator. We use a sigmoid activation at the output layer of $G_{{\boldsymbol{\theta }}_2}$ so that it takes values in [0, 1]. The network parameters $({\boldsymbol{\theta }}_1, {\boldsymbol{\theta }}_2)$ and ${\boldsymbol{\phi }}$ are estimated by solving the minimax problem:

$$\begin{aligned} (\hat{{\boldsymbol{\theta }}}_{1n},\hat{{\boldsymbol{\theta }}}_{2n}, \hat{{\boldsymbol{\phi }}}_n)=\arg \min _{{\boldsymbol{\theta }}_1, {\boldsymbol{\theta }}_2}\max _{{\boldsymbol{\phi }}}\mathcal {L}_n(G_{{\boldsymbol{\theta }}_1}, G_{{\boldsymbol{\theta }}_2}, D_{{\boldsymbol{\phi }}}). \end{aligned}$$

Noting the indicator $\Delta \in \{0, 1\}$, we define the estimated conditional generator as

$$\begin{aligned} \widehat{G}_n = (\widehat{G}_{1n}, \widehat{G}_{2n})=(G_{\hat{{\boldsymbol{\theta }}}_{1n}}, I\{ G_{\hat{{\boldsymbol{\theta }}}_{2n}}\ge 0.5 \}), \end{aligned}$$

and the estimated discriminator as $\hat{D}_n=D_{\hat{{\boldsymbol{\phi }}}_n}$.

### Stage 2: Estimation of the conditional distribution function

In the second stage, we estimate the conditional distribution functions using the existing nonparametric methods for current status data. We first describe the distribution functions based on the conditional generator *G* at the population level. We write

2 $$\begin{aligned} (Y^x, \Delta ^x)=(G_1(\eta , x), G_2(\eta , x)) \sim P_{(Y, \Delta )\mid x}, \quad x \in \mathcal {X}, \end{aligned}$$

meaning that $(Y^x, \Delta ^x)$ is distributed as the conditional distribution of $(Y, \Delta )$ given $X=x$.

We can use $\widehat{G}_n(\cdot , x)$ to generate samples that are distributed as $\widehat{P}_{(Y, \Delta )|X=x}$ and obtain Monte Carlo approximations of $\widehat{F}^x_n$, as described below. We first generate a random sample $\{\eta _j, j=1 \ldots , m\}$ that are i.i.d. distributed as $P_{\eta }$ and compute $\{\widehat{G}_n(\eta _j, x), j=1,\ldots , m\}$ for a given $x \in \mathcal {X}$, where $m \ge 1$ is a positive integer. Denote $(\widehat{Y}^x_{nj}, \widehat{\delta }^x_{nj})=\widehat{G}_n(\eta _j, x), j=1, \ldots , m.$ The NPMLE of the conditional distribution function of *T* given $X=x$ is

3 $$\begin{aligned} \widehat{F}_{nm}^x (\widehat{Y}_{(ni)}^x) = \max _{r \le i} \min _{k \ge i} \frac{ \sum _{r \le j \le k} \widehat{\delta }^x_{(nj)}}{k-r+1}, \end{aligned}$$

where $\widehat{Y}^x_{(n1)} \le \cdots \le \widehat{Y}^x_{(nm)}$ are the ordered values of $\widehat{Y}^x_{n1}, \ldots , \widehat{Y}^x_{nm}$ and $\widehat{\delta }^x_{(nj)}$ is the censoring indicator associated with $\widehat{Y}^x_{(nj)}.$

### Implementation

The proposed WGAN-based estimation procedure is implemented by two stages. In the first stage, we train the conditional generator to obtain the estimators of the neural network parameters; in the second stage, we generate samples from the conditional generator for given $X = x$ and then compute the NPMLE based on the generated samples.

Let $(G_{{\boldsymbol{\theta }}_1}, G_{{\boldsymbol{\theta }}_2})$ and $D_{\boldsymbol{\phi }}$ denote the conditional generator for $(Y,\Delta )$ and the discriminator respectively, where ${\boldsymbol{\theta }}_1, {\boldsymbol{\theta }}_2$ and ${\boldsymbol{\phi }}$ are the corresponding parameters in neural networks. The estimators $(\hat{\boldsymbol{\theta }}_1, \hat{\boldsymbol{\theta }}_2,\hat{\boldsymbol{\phi }})$ of the neural network parameters $({\boldsymbol{\theta }}_1, {\boldsymbol{\theta }}_2, {\boldsymbol{\phi }})$ are the solution to the minimax problem,

4 $$\begin{aligned} ({\boldsymbol{\theta }}_1, {\boldsymbol{\theta }}_2, {\boldsymbol{\phi }}) = \arg \min _{{\boldsymbol{\theta }}_1,{\boldsymbol{\theta }}_2}\max _{{\boldsymbol{\phi }}} \frac{1}{n}\sum _{i=1}^n\{&D_{\boldsymbol{\phi }}(X_i, G_{{\boldsymbol{\theta }}_1}(\eta _i, X_i), G_{{\boldsymbol{\theta }}_2}(\eta _i, X_i))- D_{\boldsymbol{\phi }}(X_i,Y_i, \Delta _i) \nonumber \\&-\lambda (\Vert \nabla _{(x, y)}D_{\boldsymbol{\phi }}(X_i,Y_i, \Delta _i)\Vert _2-1)^2\big \}, \end{aligned}$$

where $\nabla _{(x,y)}D_{\boldsymbol{\phi }}(X_i,Y_i, \Delta _i)$ is the gradient of $D_{\boldsymbol{\phi }}(x, y, \delta )$ with respect to (*x*, *y*) evaluated at $(X_i, Y_i, \Delta _i).$ Here we use the gradient penalty algorithm to impose the constraint that the discriminator belongs to the class of 1-Lipschitz functions (Gulrajani et al. 2017). The minimax problem (4) is solved by updating $({\boldsymbol{\theta }}_1,{\boldsymbol{\theta }}_2)$ and ${\boldsymbol{\phi }}$ alternatively as follows:

(i) At the *k*th iteration, we fix ${\boldsymbol{\theta }}_1$ and ${\boldsymbol{\theta }}_2$ at the values of the $(k-1)$th iteration, ${\boldsymbol{\theta }}_1^{[k-1]}$ and ${\boldsymbol{\theta }}_2^{[k-1]}$, and update the discriminator by maximizing the empirical objective function,
  $$\begin{aligned} {\boldsymbol{\phi }}^{[k]} =&\mathop {\textrm{argmax}}\limits _{{\boldsymbol{\phi }}} \frac{1}{n}\sum _{i=1}^n\{ D_{\boldsymbol{\phi }}(X_i, G_{{\boldsymbol{\theta }}_1^{[k-1]}}(\eta _i, X_i), G_{{\boldsymbol{\theta }}_2^{[k-1]}}(\eta _i, X_i)) \\&-D_{\boldsymbol{\phi }}(X_i,Y_i, \Delta _i) -\lambda (\Vert \nabla _{(x, y)}D_{\boldsymbol{\phi }}(X_i,Y_i, \Delta _i)\Vert _2-1)^2\big \} \end{aligned}$$
  with respect to ${\boldsymbol{\phi }}.$ The third term on the right side is the penalty imposed on the gradient norm of the discriminator. Recall that $D\in \mathcal {F}^1_\textrm{Lip}$, hence its gradient norm is at most 1 everywhere. Following (Gulrajani et al. 2017), we construct this two-sided penalty that pushes the gradient norm towards 1 instead of below 1 (one-sided penalty).
(ii) Fixing ${\boldsymbol{\phi }}$ at ${\boldsymbol{\phi }}^{[k]}$, we update the generator by minimizing the empirical objective function,
  $$\begin{aligned} (\hat{\boldsymbol{\theta }}_1^{[k]}, \hat{\boldsymbol{\theta }}_2^{[k]})= \mathop {\textrm{argmin}}\limits _{{\boldsymbol{\theta }}_1,{\boldsymbol{\theta }}_2}\frac{1}{n}\sum _{i=1}^n D_{{\boldsymbol{\phi }}^{[k]}}(X_i, G_{{\boldsymbol{\theta }}_1}(\eta _i, X_i), G_{{\boldsymbol{\theta }}_2}(\eta _i, X_i)). \end{aligned}$$
  The conditional generator $G_1$ for the observed time *Y* has a linear output activation function. The conditional generator $G_2$ for the indicator has a sigmoid output activation function, then the status is determined based on whether the value of the sigmoid function is greater than 0.5 or not. The two generators $G_1$ and $G_2$ can have the same structure and share the same parameters except for the output layer. This method is described in Algorithm 1 and implemented using TensorFlow (Abadi et al. 2016).

**Algorithm 1:**
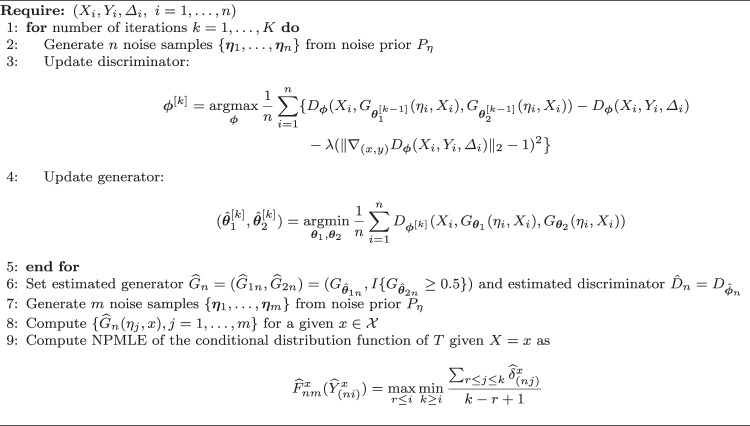
Generative conditional distribution estimation

## Asymptotic theory

For simplicity, we write the generator $G=(G_1, G_2)$, and the generator network $G_{{\boldsymbol{\theta }}}=(G_{{\boldsymbol{\theta }}_{1}}, G_{{\boldsymbol{\theta }}_{2}})$. Let $H^x$ and $H_{1}^x$ be the distribution functions of $Y^x$ and $(Y^x, \Delta ^x=1)$, respectively,

5 $$\begin{aligned} H^x(t)=P(Y^x\le t), \quad H^x_{1}(t)=P( Y^x\le t, \Delta ^x=1). \end{aligned}$$

According to $H^x_1(t)=\int ^t_0 F^x(u)dH^x(u)$, the conditional distribution $F^x$ can be uniquely derived as

6 $$\begin{aligned} F^x=\frac{dH^x_1}{dH^x}, \quad a.e. \text { with the measure induced by} H^x. \end{aligned}$$

We estimate the conditional distribution functions as follows. Let $(\widehat{G}_{1n}, \widehat{G}_{2n})$ be the estimated conditional generator. For any $x\in \mathcal {X}$, the distribution of $(\widehat{G}_{1n}(\eta , x), \widehat{G}_{2n}(\eta , x))$ is an estimator of the conditional distribution $P_{(T,\Delta )\mid X=x}.$ Similar to (2), we write $(\widehat{Y}^x, \hat{\Delta }^x)=(\widehat{G}_{1n}(\eta ,x), \widehat{G}_{2n}(\eta , x)).$ Let

7 $$\begin{aligned} \widehat{H}^x_n(t)=P(\widehat{Y}^x\le t), \quad \widehat{H}^x_{1n}(t)=P(\widehat{Y}^x\le t, \widehat{\Delta }^x=1). \end{aligned}$$

Corresponding to (6) it is natural to consider the Radon–Nikodym derivative $d\widehat{H}^x_{1n}/d\widehat{H}^x_n$ as an estimator of $F^x$. However, this may not be a reasonable choice, because $d\widehat{H}^x_{1n}/d\widehat{H}^x_n$ is not guaranteed to be a distribution or a subdistribution. To construct a valid estimator $\widehat{F}_n^x$, we maximize the log-likelihood $l_n(F)$ over the class of all distribution functions, where

$$\begin{aligned} \begin{aligned} l_{n}(F) = \int \log F(u)d\widehat{H}_{1n}^x(u)+\int \log (1-F( u))d\widehat{H}_{0n}^x(u), \end{aligned} \end{aligned}$$

with $\widehat{H}_{0n}^x(u)=\widehat{H}_{n}^x(u)-\widehat{H}_{1n}^x(u).$ The estimator $\widehat{F}_n^x$ is guaranteed to be a distribution, which is a uniformly consistent estimator of $F^x$ as shown below. To study the properties of the proposed method, we impose some assumptions.

### Assumption 1

For some $\gamma>0$, (*X*, *Y*) satisfies

$$\begin{aligned} \mathbb {E}\Vert (X, Y)\Vert 1_{\{\Vert (X, Y)\Vert>\log t\}}=O\left( t^{-(\log t)^\gamma /(d+2)}\right) , \end{aligned}$$

for any $t \ge 1$, where $\Vert \cdot \Vert$ denotes the Euclidean norm.

### Assumption 2

The noise distribution $P_{\eta }$ is absolutely continuous with respect to the Lebesgue measure.

Let $P_{F^x}$ and $P_{H^x}$ denote the probability measures induced by $F^x$ and $H^x$, respectively.

### Assumption 3

For every $x\in \mathcal {X}$, $T^x$ and $Y^x$ have continuous distributions $F^x$ and $H^x$, satisfying $P_{F^x}$ is absolutely continuous with respect to $P_{H^x}.$

Assumption 1 is satisfied if $P_{X,Y}$ is subgaussian. Assumption 2 is satisfied by commonly used reference distributions such as normal and uniform distributions. Assumption 3 is a technical condition.

For a matrix $V=(v_{i,j}),$ let $\Vert V\Vert _{\infty }=\sup _{\Vert x\Vert _{\infty }=1}\Vert Vx\Vert _{\infty }$ be the $\infty$-norm of *V*. For the conditional generator, define $K({\boldsymbol{\theta }}_i)=\Vert (V_{i,L_1},b_{i,L_1})\Vert _{\infty }\prod _{j=0}^{L_1-1}\max \{\Vert (V_{i,j},b_{i,j})\Vert _{\infty },1\}$ for $i=1,2.$ For the discriminator, define $K({\boldsymbol{\phi }})= \Vert (V_{L_2},b_{L_2})\Vert _{\infty }\prod _{j=0}^{L_2-1}\max \{\Vert (V_{j},b_{j})\Vert _{\infty },1\}$.

### Theorem 1

Suppose Assumptions 1–3 hold. Let $(L_1, W_1)$ of $G_{{\boldsymbol{\theta }}}$ be specified such that $W_1^2 L_1= c n$ for some constants $24\le c\le 768$. Suppose that the conditional generator satisfies $\Vert G_{ {\boldsymbol{\theta }}_1}\Vert _{\infty } \le \log n$ and $K({\boldsymbol{\theta }}_i)\le K_1$, $i=1,2,$ for some positive constant $K_1$. Suppose that the discriminator satisfies $K({\boldsymbol{\phi }})\le \log n K_2$. Let $\gamma _2=\lceil \log _2 (d+2)\rceil$. There exists $c_2>0$ such that for any $W_2\ge c_2(K_2/\log ^{\gamma _2}K_2)^{(2d+5)/(2d+6)}$ and $L_2\ge 4\gamma _2+2$, if we select $K_2\asymp n^{(d+3)/(d+2)}$, then we have $\sup _{t\in \mathbb {R}} \left| \widehat{F}_{n}^X(t)-F^X(t)\right| {\mathop {\rightarrow }\limits ^\mathcal{P}} 0$ as $n\rightarrow \infty .$

### Theorem 2

Under the same assumptions and conditions of Theorem 1, we have

$$\sup _{t\in \mathbb {R}} \left| \widehat{F}_{nm}^X(t)-F^X(t)\right| {\mathop {\rightarrow }\limits ^\mathcal{P}} 0, \quad \text{ as } \quad n, m\rightarrow \infty .$$

The theorem guarantees that the estimator of the conditional cumulative distribution function constructed by the proposed two-stage estimation method is consistent.

## Simulation studies

We conducted simulation studies under four different settings to evaluate the finite sample performance of GCDE and compared it with the Cox proportional hazards model and the accelerated failure time (AFT) model. For all four scenarios detailed below, we set sample size $n=5,000$ and considered $X \sim N(\textbf{0}, \textbf{I}_6)$ and examination time $C \sim Uniform(0, \tau )$, with $\tau$ chosen to achieve a censoring rate of 40%.

Model 1: Assume a proportional hazards model with a constant baseline hazard, where $\lambda (t|x)=\lambda _0 \exp (\beta (x))$, with $\beta (x) =x_1-0.5x_2-1.5x_3+2x_4-0.3x_5-x_6$ and $\lambda _0=1$.

Model 2: Assume that *T* given $X=x$ follows a Weibull distribution with the conditional survival function $S(t|x)= \exp [-(t/\psi (x))^\nu ]$, and the shape parameter $\nu =2$ and the scale parameter $\psi (x)=\exp (1.2x_1 x_2 - 0.7x_3 x_4 + x_5 - x_6)$.

Model 3: Assume an AFT model with a normally distributed error: $\log (T) = 0.5x_1 - 1.1x_2 + 0.8x_3 - x_4 + x_5 - 0.3x_6 + \epsilon$, with $\epsilon \sim \text{ Normal }(0,0.5)$.

Model 4: Assume an AFT model with both linear and nonlinear covariate effects and an exponentially distributed errors: $\log (T) = 0.5x_1^2 x_2^2 -0.8x_3 + x_4 - 0.7x_5 + 0.5x_6 +\epsilon$, with $\exp (\epsilon ) \sim \text{ Exponential }(1)$.

The sample consists of $\{(X_i, Y_i, \Delta _i), i=1, \ldots , n\}$, where $Y_i$ is the natural logarithm of examination time $C_i$ and $\Delta _i=I(T_i \le C_i)$. We implemented a two-layer feedforward neural network for both the generator and discriminator. We used (60, 30) nodes for both the generator and discriminator under Model 1; (60, 30) nodes for the generator and (40, 20) nodes for the discriminator under Model 2; (40, 20) nodes for the generator and (30, 15) nodes for the discriminator under Model 3; and (40, 20) nodes for both the generator and discriminator under Model 4. We adopted the ReLU activation function for both of the hidden layers. For Models 1–4, we considered noise vectors $\eta$ of length 10, and $\tau =3, 4, 5, 5$, respectively. We assumed $\eta \sim \text{ Normal }(0,I)$ and $m=10,000$. The *R* package *curstatCI* was applied to obtain NPMLEs based on the conditional samples generated by the proposed method. For comparison, we provided the corresponding simulation results under the Cox model based on the data sets generated from Models 1 and 2 and those under the AFT model with normal errors based on the data sets generated from Models 3 and 4. Distribution functions under the Cox and AFT models were estimated using the *R* package *icenReg*.

**Fig. 1:**
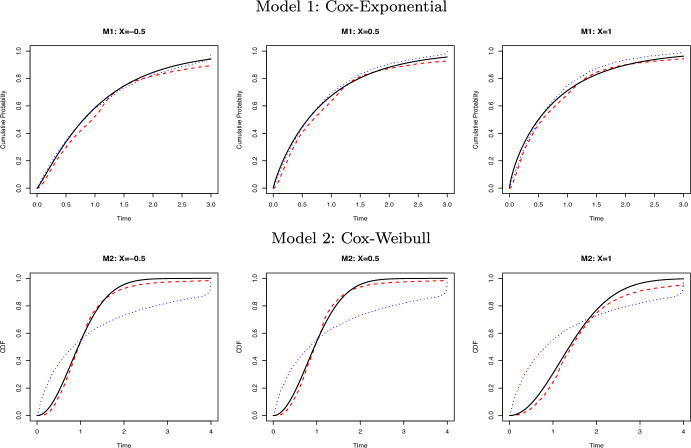
Comparison of the true conditional cumulative distribution function (solid black line) with the estimated ones using the GCDE (red dashed line) and the Cox model (blue dotted line) given $x=(\mathbf{-0.5}_6)^\top , (\mathbf{0.5}_6)^\top , (\mathbf{1.0}_6)^\top$ respectively

**Fig. 2:**
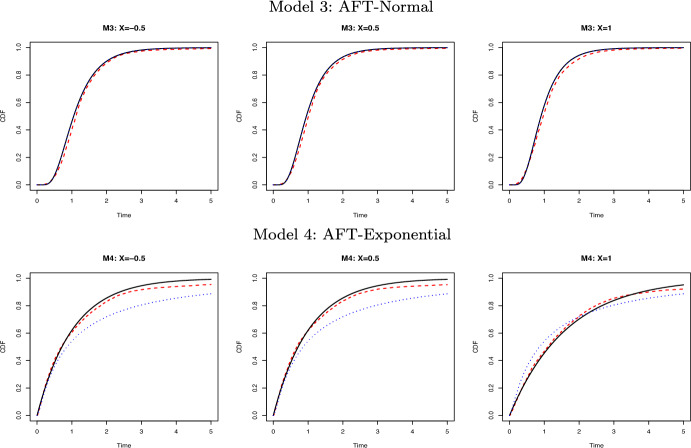
Comparison of the true conditional cumulative distribution function (solid black line) with the estimated ones using the GCDE (red dashed line) and the AFT model (blue dotted line) given $x=(\mathbf{-0.5}_6)^\top , (\mathbf{0.5}_6)^\top , (\mathbf{1.0}_6)^\top$ respectively

We fixed the covariate vector $x=\{(-\mathbf{0.5}_6)^\top , (\mathbf{0.5}_6)^\top , (\mathbf{1.0}_6)^\top \}$, with each vector of length 6, and replicated 200 Monte Carlo simulations. In the Appendix, Tables 1 and 2 summarize the bias and standard deviation (SD) of the conditional cumulative distribution function evaluated at 10 equally spaced time points from 0 to $\tau$. Figures 1 and 2 compare the true distribution function with estimates obtained by the GCDE method, the Cox model, and the AFT model, respectively. The GCDE method and the Cox model yielded similar results under Model 1, while under Model 2 the GCDE method evidently outperformed the Cox model when the proportional hazards assumption was violated. Under Model 3, the AFT model provided nearly perfect estimates, which is unsurprising because the fitted model is correct. The GCDE method also yielded reasonably small bias and SD. Under Model 4, the AFT model was unable to capture the nonlinearity in the true model, while the GCDE method consistently showed its good performance.

**Table 1:** The conditional cumulative distribution function evaluated at different time points given $x=(\mathbf{-0.5}_6)^\top , (\mathbf{0.5}_6)^\top , (\mathbf{1.0}_6)^\top$, respectively, based on 200 simulations

*x*		Model 1: Cox-exponential									
		0.3	0.6	0.9	1.2	1.5	1.8	2.1	2.4	2.7	3.0
		GCDE									
$-$0.5	Bias	$-$0.0286	$-$0.0458	$-$0.0672	$-$0.0205	0.0004	$-$0.0152	$-$0.0308	$-$0.0403	$-$0.0455	$-$0.0494
	SD	0.0054	0.0063	0.0065	0.0050	0.0047	0.0043	0.0044	0.0048	0.0051	0.0053
0.5	Bias	$-$0.0556	$-$0.0452	$-$0.0493	$-$0.0082	0.0087	$-$0.0011	$-$0.0134	$-$0.0205	$-$0.0255	$-$0.0287
	SD	0.0061	0.0065	0.0063	0.0044	0.0038	0.0034	0.0035	0.0037	0.0039	0.0040
1	Bias	$-$0.0498	$-$0.0360	$-$0.0354	0.0001	0.0165	0.0084	$-$0.0017	$-$0.0099	$-$0.0170	$-$0.0190
	SD	0.0085	0.0077	0.0072	0.0049	0.0034	0.0029	0.0031	0.0032	0.0033	0.0034
		Cox									
$-$0.5	Bias	0.0191	0.0064	$-$0.0079	$-$0.0188	$-$0.0156	$-$0.0184	$-$0.0213	$-$0.0197	$-$0.0139	0.0514
	SD	0.0028	0.0031	0.0034	0.0030	0.0026	0.0026	0.0021	0.0020	0.0017	0.0011
0.5	Bias	$-$0.0168	$-$0.0001	0.0110	0.0165	0.0264	0.0254	0.0216	0.0195	0.0188	0.0407
	SD	0.0036	0.0035	0.0033	0.0028	0.0024	0.0020	0.0015	0.0012	0.0011	0.0005
1	Bias	$-$0.0315	0.0040	0.0254	0.0347	0.0437	0.0409	0.0348	0.0301	0.0263	0.0358
	SD	0.0043	0.0041	0.0036	0.0031	0.0025	0.0021	0.0015	0.0011	0.0009	0.0003

*x*		Model 2: Cox-weibull									
		0.4	0.8	1.2	1.6	2.0	2.4	2.8	3.2	3.6	4.0
		GCDE									
$-$0.5	Bias	$-$0.0411	$-$0.0297	0.0259	$-$0.0048	$-$0.0294	$-$0.0328	$-$0.0283	$-$0.0234	$-$0.0197	$-$0.0158
	SD	0.0056	0.0124	0.0111	0.0072	0.0045	0.0029	0.0023	0.0022	0.0021	0.0019
0.5	Bias	$-$0.0404	$-$0.0242	0.0433	0.0141	$-$0.0188	$-$0.0274	$-$0.0262	$-$0.0225	$-$0.0190	$-$0.0158
	SD	0.0062	0.0126	0.0103	0.0061	0.0040	0.0026	0.0021	0.0019	0.0017	0.0016
1	Bias	$-$0.0259	$-$0.0597	$-$0.0355	$-$0.0190	$-$0.0246	$-$0.0432	$-$0.0529	$-$0.0570	$-$0.0526	$-$0.0461
	SD	0.0043	0.0103	0.0156	0.0144	0.0113	0.0086	0.0065	0.0056	0.0047	0.0044
		Cox									
$-$0.5	Bias	0.2143	0.0980	$-$0.0707	$-$0.1884	$-$0.2259	$-$0.2139	$-$0.1914	$-$0.1643	$-$0.1418	$-$0.0099
	SD	0.0025	0.0024	0.0024	0.0021	0.0020	0.0019	0.0018	0.0017	0.0015	0.0020
0.5	Bias	0.2133	0.0968	$-$0.0719	$-$0.1896	$-$0.2272	$-$0.2151	$-$0.1925	$-$0.1653	$-$0.1428	$-$0.0099
	SD	0.0025	0.0024	0.0024	0.0022	0.0020	0.0019	0.0018	0.0017	0.0016	0.0020
1	Bias	0.2731	0.2791	0.1904	0.0634	$-$0.0427	$-$0.1070	$-$0.1397	$-$0.1433	$-$0.1353	$-$0.0073
	SD	0.0027	0.0027	0.0027	0.0026	0.0024	0.0023	0.0022	0.0020	0.0019	0.0020

**Table 2:** The conditional cumulative distribution function evaluated at at different time points given $x=(\mathbf{-0.5}_6)^\top , (\mathbf{0.5}_6)^\top , (\mathbf{1.0}_6)^\top$, respectively, based on 200 simulations

*x*		Model 3: AFT-normal									
		0.5	1.0	1.5	2.0	2.5	3.0	3.5	4.0	4.5	5.0
		GCDE									
$-$0.5	Bias	$-$0.0085	$-$0.0582	$-$0.0262	$-$0.0129	$-$0.0061	$-$0.0082	$-$0.0089	$-$0.0088	$-$0.0079	$-$0.0062
	SD	0.0045	0.0100	0.0069	0.0043	0.0025	0.0016	0.0012	0.0010	0.0009	0.0008
0.5	Bias	$-$0.0137	$-$0.0489	$-$0.0189	$-$0.0166	$-$0.0089	$-$0.0077	$-$0.0077	$-$0.0070	$-$0.0061	$-$0.0052
	SD	0.0058	0.0098	0.0061	0.0039	0.0020	0.0012	0.0009	0.0009	0.0008	0.0007
1	Bias	0.0010	$-$0.0520	$-$0.0328	$-$0.0257	$-$0.0138	$-$0.0102	$-$0.0079	$-$0.0064	$-$0.0052	$-$0.0043
	SD	0.0082	0.0126	0.0076	0.0042	0.0025	0.0016	0.0011	0.0009	0.0008	0.0008
		AFT									
$-$0.5	Bias	0.0000	0.0019	0.0028	0.0020	0.0011	0.0005	0.0002	0.0001	0.0000	0.0000
	SD	0.0009	0.0018	0.0014	0.0009	0.0005	0.0003	0.0002	0.0001	0.0000	0.0000
0.5	Bias	$-$0.0007	0.0003	0.0014	0.0010	0.0005	0.0002	0.0001	0.0000	0.0000	0.0000
	SD	0.0012	0.0017	0.0011	0.0006	0.0003	0.0002	0.0001	0.0000	0.0000	0.0000
1	Bias	$-$0.0005	$-$0.0008	0.0001	0.0001	0.0000	$-$0.0000	$-$0.0000	$-$0.0000	$-$0.0000	$-$0.0000
	SD	0.0017	0.0028	0.0017	0.0008	0.0004	0.0002	0.0001	0.0000	0.0000	0.0000

*x*		Model 4: AFT-exponential									
		0.5	1.0	1.5	2.0	2.5	3.0	3.5	4.0	4.5	5.0
		GCDE									
$-$0.5	Bias	0.0179	$-$0.0139	$-$0.0260	$-$0.0265	$-$0.0233	$-$0.0290	$-$0.0360	$-$0.0398	$-$0.0404	$-$0.0376
	SD	0.0087	0.0083	0.0060	0.0050	0.0037	0.0031	0.0028	0.0027	0.0027	0.0028
0.5	Bias	0.0218	$-$0.0026	$-$0.0224	$-$0.0229	$-$0.0213	$-$0.0246	$-$0.0325	$-$0.0377	$-$0.0397	$-$0.0394
	SD	0.0079	0.0078	0.0058	0.0045	0.0035	0.0031	0.0028	0.0028	0.0028	0.0027
1	Bias	0.0099	0.0128	0.0156	0.0216	0.0233	0.0158	0.0013	$-$0.0114	$-$0.0206	$-$0.0301
	SD	0.0088	0.0094	0.0084	0.0069	0.0056	0.0049	0.0043	0.0043	0.0043	0.0043
		AFT									
$-$0.5	Bias	$-$0.0294	$-$0.0794	$-$0.1175	$-$0.1370	$-$0.1430	$-$0.1407	$-$0.1337	$-$0.1247	$-$0.1150	$-$0.1054
	SD	0.0013	0.0011	0.0010	0.0009	0.0009	0.0008	0.0008	0.0007	0.0007	0.0007
0.5	Bias	$-$0.0295	$-$0.0795	$-$0.1176	$-$0.1371	$-$0.1431	$-$0.1407	$-$0.1338	$-$0.1247	$-$0.1150	$-$0.1054
	SD	0.0014	0.0012	0.0010	0.0009	0.0008	0.0007	0.0007	0.0007	0.0006	0.0006
1	Bias	0.0931	0.0862	0.0511	0.0160	$-$0.0125	$-$0.0335	$-$0.0480	$-$0.0574	$-$0.0628	$-$0.0654
	SD	0.0019	0.0018	0.0016	0.0014	0.0013	0.0012	0.0011	0.0010	0.0009	0.0009

Table 3 below presents the computation times for simulations and the application are reported in minutes, averaged over five replications. The lengths of computation time vary across different simulation setups due to the varying complexity of the model and data. All computations were performed on a MacBook Pro laptop equipped with an Apple M1 Max chip and 64 GB of memory. The programming language used for the implementation was R.

**Table 3:** The computation times for the simulation studies, including Model 1 (M1), Model 2 (M2), Model 3 (M3), and Model 4 (M4), as well as the data application (Data), are reported in minutes, averaged over five replications

	M1	M2	M3	M4	Data
Time (min)	13.286	27.805	18.040	17.956	30.717

## Data example

We applied the proposed method to analyzing the seroprevalence data on Parvovirus B19, which is an infectious agent of erythema infectiosum, also referred to as slapped cheek syndrome or fifth disease (Broliden et al. 2006). Such disease is more common amongst children and causes mild symptoms including fever, runny nose, headache and rash. Between 1995 and 2004, the study investigated 13,449 serum samples from large national serum banks in Belgium, Finland, England & Wales, Italy, and Poland. Collected sera covered age up to 82 years and enzyme immunoassay testing was performed to identify presence of immunoglobulin G antibody with results as either seropositive or seronegative. Seropositive refers to confirmed presence of the IgG antibody and seronegative means otherwise. Details of the study can be found in Mossong et al. (2008). We treated age as the examination time and IgG antibody test results as the event outcome, such that seropositive results were considered as event. Hence, the infection status at the examination time was known but the exact time when patients contracted the Parvovirus B19 virus remained unknown.

After removing erroneous records and missing entries, data on 6,625 subjects remained for analysis with an overall censoring rate 44.5% and males proportion 47.1%. We allocated 90% of the sample to training (5,692 subjects) and 10% to testing (663 subjects), with the training set intended for learning the model and the testing set for validating prediction accuracy. The censoring rates among testing and training sets were 44.3% and 46.5%, respectively. We considered two covariates including gender as a binary covariate (1 for male and 0 for female) and level of IgG antibody concentration against Parvovirus B19 (in ml) as a continuous covariate. Both covariates were standardized to yield zero mean and unit variance and time was log transformed prior to training the model. Event outcome was immunological status of subjects, with 1 indicating presence of Parvovirus B19 virus and 0 otherwise. We implemented the proposed GCDE with a two-layer feedforward neural network containing (40, 20) nodes for both the generator and discriminator. For the two hidden layers, we adopted the ReLU activation function. We considered noise vector $\eta$ of length 20 and assumed $\eta \sim \text{ Normal }(0,I)$ with $m=10,000$.

**Fig. 3:**
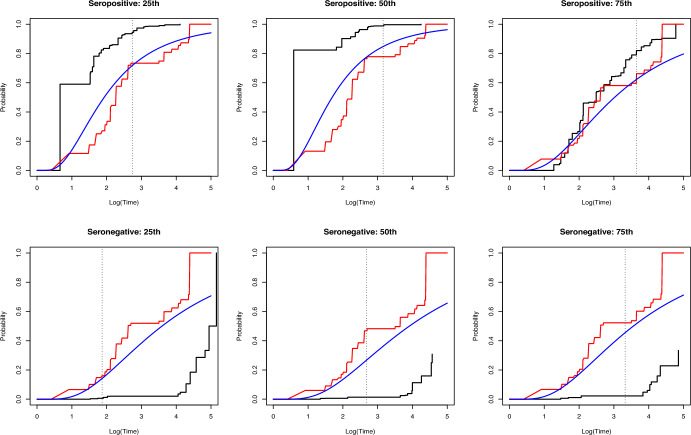
Comparison of the conditional cumulative distribution functions estimated using the GCDE (black solid line), the Cox model (red solid line) and the AFT model (blue solid line) with normal errors for subjects with examination times at the 25*th*, 50*th* and 75*th* percentiles in the seropositive and the seronegative subpopulations, respectively. Vertical dotted grey lines represent the examination times for those patients

Conditional samples were generated for patients in the testing set and a subject was classified seropositive when more than half of the generated sample was predicted to be seropositive. Of the 308 seronegative subjects in the testing set, 283 were correctly predicted, and for the remaining 355 subjects who were tested seropositive, 19 were incorrectly predicted as seronegative. This leads to an overall accuracy rate of 93.4%. Next, we apply the NPMLE to estimate the cumulative distribution function (CDF) of the infected time for Parvovirus B19 virus based on conditional samples. Figure 3 shows the estimated CDF for three seronegative and three seropositive subjects from the testing set, with observation times corresponding to 25*th*, 50*th* and 75*th* percentiles. Intuitively, the CDF should be extremely low up to the examination time for subjects whose infection was not observed, while it should be relatively high for infected patients. Hence, the proposed method yielded high prediction accuracy as well as reasonable estimates for the CDF.

**Fig. 4:**
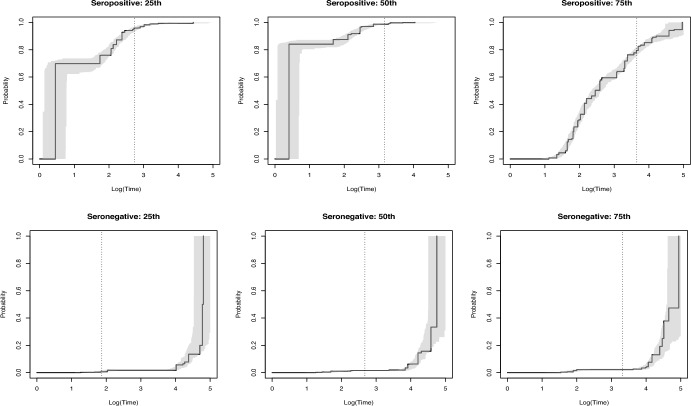
Grey regions represent the estimated distribution function with 95% confidence intervals for subjects with examination times at the 25*th*, 50*th* and 75*th* percentiles in the seropositive and the seronegative subpopulations. Grey dotted lines represent the examination times for those patients

In applications, confidence intervals play a crucial role in assessing the uncertainty associated with estimators. To construct 95% confidence intervals for the proposed estimator, we performed the following steps. We repeated Stage 2 of the proposed estimation process *M* times (*M* is a large number), resulting in *M* estimates. Next, we calculated the lower bound of the confidence interval as the 2.5*th* percentile of the estimates, and the upper bound as the 97.5*th* percentile. The grey shaded region in Fig. 4 represents the confidence interval for patients from the testing dataset at the 25*th*, 50*th* and 75*th* percentiles of survival times (three seropositive and three seronegative patients) with $M=100$, $m=10,000$, and dimension of $\eta$ equal to 20.

## Concluding remarks

We propose a two-stage generative approach to analyzing current status data, which combines advantages of recently developed generative learning and nonparametric estimation. The first stage learns a conditional generator for the joint distribution of the observation time and binary status indicator, and then the second stage applies the NPMLE to samples from the conditional generator to estimate the conditional distribution of failure time given predictors. The proposed method is model-free and hence does not suffer from model misspecification as the existing methods. As shown by simulation scenarios 2 and 4, where nonlinearity exists in the covariates, our proposed method consistently perform well while the Cox and AFT models suffer from large bias due to model misspecifications. The GAN-based estimation procedure provides a novel and robust approach to statistical inference about conditional distributions of survival times given predictors.

This work presents significant advancements in the analysis of survival data by leveraging the power of machine learning. The innovative approaches presented in this paper offer valuable insights and contribute to the growing field of survival analysis, highlighting the beauty and potential of machine learning in this domain. Survival analysis is an important field in biostatistics and medical data analysis, as many clinical trials and drug development rely upon skills in this field and the treatments evidenced by survival analysis can save millions of patients’ lives.

The training process for our model is time-consuming due to factors such as data complexity, WGAN instability, and hyperparameter tuning. Another limitation of our approach is that it does not provide direct interpretations of the covariates in terms of their effects on the outcome. However, we can develop extensions for estimating treatment effects by including a semiparametric component in the proposed framework. For future studies, it would be interesting to expand the current theoretical framework and investigate the asymptotic distribution of the estimator. Furthermore, extensions based on the GCDE to accommodate the general type of interval-censored data also warrant further research.

## Appendix

### Lemmas

Let $\Omega$ be the sample space of $(X,Y,\Delta )$ and let $\mu$ be the equipped probability. Let $\Lambda$ be the sample space of $\eta$ and let $\nu$ be the equipped probability. Let $\Vert f\Vert _{\infty }=\sup _{t\in \mathbb {R}}|f(t)|$ denote the supremum norm of function *f*. Denote $\widetilde{G}_n = (G_{\hat{{\boldsymbol{\theta }}}_{1n}}, G_{\hat{{\boldsymbol{\theta }}}_{2n}}).$ The following consistency result for the conditional generator is given by Zhou et al. (2022).

#### Lemma 1

Suppose that Assumptions 1 and 2 hold. Let $(L_1, W_1)$ of $G_{{\boldsymbol{\theta }}}$ be specified such that $W_1^2 L_1= c n$ for some constants $24\le c\le 768$. Suppose that the conditional generator satisfies $\Vert G_{ {\boldsymbol{\theta }}_1}\Vert _{\infty } \le \log n$ and $K({\boldsymbol{\theta }}_i)\le K_1$, $i=1,2,$ for some positive constant $K_1$. Suppose that the discriminator satisfies $K({\boldsymbol{\phi }})\le \log n K_2$. Let $\gamma _2=\lceil \log _2 (d+2)\rceil$. There exists $c_2>0$ such that for any $W_2\ge c_2(K_2/\log ^{\gamma _2}K_2)^{(2d+5)/(2d+6)}$ and $L_2\ge 4\gamma _2+2$, if we select $K_2\asymp n^{(d+3)/(d+2)}$, then

A.1 $$\begin{aligned} d_{\mathcal {H}^1_B}(P_{\widetilde{G}_n(\eta ,X)}, P_{(Y,\Delta )|X})=\sup _{h\in \mathcal {H}^1_B}\mathbb {E}_{\eta }(h(\widetilde{G}_n(\eta , X)))-\mathbb {E}(h(Y,\Delta )|X)\rightarrow _{P_{\widetilde{G}_n, X}} 0, \end{aligned}$$

where $P_{\widetilde{G}_n, X}$ represents the probability with respect to the randomness in $\widetilde{G}_n$ and *X*.

The consistency of $\widehat{H}^x_n$ and $\widehat{H}^x_{1n}$ follows from Zhou et al. (2022).

#### Lemma 2

Suppose that Assumption 3 holds. Under the same assumptions and conditions of Lemma 1, we have $\Vert \widehat{H}^X_n(t)- H^X(t)\Vert _{\infty } {\mathop {\rightarrow }\limits ^\mathcal{P}} 0 \ \text { and } \Vert \widehat{H}^X_{1n}(t)- H^X_1(t)\Vert _{\infty } {\mathop {\rightarrow }\limits ^\mathcal{P}} 0.$

### Proof of Theorem 1

#### Proof

From Lemma 2, for every subsequence $\Vert \widehat{H}^X_{n_k}- H^X\Vert _{\infty }$ or $\Vert \widehat{H}^X_{1n_k}- H^X_1 \Vert _{\infty }$, there is a further subsequence $\Vert \widehat{H}^X_{n_{k_{l}}}- H^X\Vert _{\infty }$ or $\Vert \widehat{H}^X_{1n_{k_{l}}}- H^X_1 \Vert _{\infty }$ that converges almost surely to 0. For the simplicity of notation, we denote the subsequences by $\Vert \widehat{H}^X_{n_{k}}- H^X\Vert _{\infty }$ and $\Vert \widehat{H}^X_{1n_{k}}- H^X_1\Vert _{\infty }$. Define

$$\Omega _1=\left\{ \omega \in \Omega : \lim _{k\rightarrow \infty }\Vert \widehat{H}^X_{n_{k}}- H^X\Vert _{\infty }=0 \text { and }\lim _{k\rightarrow \infty }\Vert \widehat{H}^X_{1n_{k}}- H^X_1\Vert _{\infty }=0\right\} ,$$

so $\mu (\Omega _1)=1$. For a fixed $\omega \in \Omega _1$, let $x=X(\omega )$ and $H^x_{0}=H^x-H^x_{1}$. Then, we have

A.2 $$\begin{aligned} \lim _{k\rightarrow \infty }\Vert \widehat{H}^x_{0n_{k}}- H^x_{0}\Vert _{\infty }=0 \text { and } \lim _{k\rightarrow \infty }\Vert \widehat{H}^x_{1n_{k}}- H^x_1\Vert _{\infty } =0. \end{aligned}$$

Consider the subsequence $\{\widehat{F}_{n_{k}}^x\}$. By its definition, we have, for any $\varepsilon \in (0,1)$,

$$l_{n_{k}}((1-\varepsilon )\widehat{F}^x_{n_{k}}+\varepsilon F^x)-l_{n_{k}}(\widehat{F}^x_{n_{k}})\le 0.$$

Therefore, $\lim _{\varepsilon \rightarrow 0^{+}}\varepsilon ^{-1}\{l_{n_{k}}((1-\varepsilon )\widehat{F}^x_{n_{k}}+\varepsilon F^x)-l_{n_{k}}(\widehat{F}^x_{n_{k}})\}\le 0$, from which we have

A.3 $$\begin{aligned} \begin{aligned} \int \frac{F^x(u) }{\widehat{F}_{n_{k}}^x(u)} d\widehat{H}_{1{n_{k}}}^x(u)+ \int \frac{1- F^x(u)}{1-\widehat{F}_{{n_{k}}}^x(u)} d\widehat{H}_{0{n_{k}}}^x(u)\le 1. \end{aligned} \end{aligned}$$

For $\delta \in (0,{1}/{2})$, we select *a*, *b* such that $F^x(a)=\delta$ and $F^x(b)=1-\delta .$ From (A.3), we have

$$\int _{(0,a]}\frac{F^x(u)}{\widehat{F}_{{n_{k}}}^x(a)}d\widehat{H}_{1{n_{k}}}^x(u)\le \int _{(0,a]}\frac{F^x(u)}{\widehat{F}_{{n_{k}}}^x(u)}d\widehat{H}_{1{n_{k}}}^x(u)\le 1.$$

It follows $\widehat{F}_{{n_{k}}}^x(a)\ge \int _{(0,a]} F^x d\widehat{H}_{1{n_{k}}}^x$, where the right side converges to $\int _{(0,a]} F^xdH_{1}^x$. Therefore, $1/\widehat{F}_{{n_{k}}}^x(u)$ is bounded for $u\in [a,b]$ when *k* is large. Similarly, we show $1/(1-\widehat{F}_{{n_{k}}}^x(u))$ is bounded for $u\in [a,b]$ when *k* is large.

By Helly’s lemma (van der Vaart 1998, Lemma 2.5), the sequence $\{\widehat{F}_{n_{k}}^x\}$ has a subsequence $\{\widehat{F}_{n_{k_{s}}}^x\}$ converging to a nondecreasing right continuous function $F^x_1$ at each continuity point of $F^x_1$. Following a similar method given in Lemma 4.1 of Groeneboom and Wellner (1992), we have

A.4 $$\begin{aligned} \begin{aligned} \lim _{s\rightarrow \infty } \int _{[a,b]} \frac{F^x }{\widehat{F}_{n_{k_{s}}}^x} d\widehat{H}_{1n_{k_{s}}}^x+ \int _{[a,b]} \frac{1- F^x}{1-\widehat{F}_{n_{k_{s}}}^x} d\widehat{H}_{0n_{k_{s}}}^x=\int _{[a,b]} \frac{F^x }{F_{1}^x} d H_{1}^x+ \int _{[a,b]} \frac{1- F^x}{1-F_1^x} dH_0^x. \end{aligned} \end{aligned}$$

By (A.3, A.4) and the monotone convergence theorem, we have

A.5 $$\begin{aligned} \begin{aligned} \lim _{a\rightarrow 0^+,b\rightarrow \infty } \int _{[a,b]} \frac{F^x }{ F_{1}^x} d H_{1}^x+ \int _{[a,b]} \frac{1- F^x}{1-F_1^x} dH_0^x&=\int \frac{F^x}{ F_{1}^x}d H_{1}^x + \frac{1- F^x}{1-F_1^x} dH_0^x\\&= \int \frac{(F^x)^2 }{ F_{1}^x} + \frac{(1- F^x)^2}{1-F_1^x} dH^x\le 1. \end{aligned} \end{aligned}$$

For fixed *t* such that $F^x(t)\in (0,1)$,

$$\begin{aligned} \frac{F^x(t)^2}{y}+\frac{(1-F^x(t))^2}{1-y}\ge 1 \end{aligned}$$

holds for any $y\in (0,1)$ and the equality holds only when $y=F^x(t)$. By the continuity of $F^x$ and the absolute continuity of $P_{F^x}$ with respect to $P_{H^x}$, we have

$$\int \frac{(F^x)^2 }{ F_{1}^x} + \frac{(1- F^x)^2}{1-F_1^x} dH^x> 1$$

when $F_{1}^x\ne F^x.$ This together with (A.5) demonstrates $F_1^x=F^x$. Therefore, we show the subsequence $\{\widehat{F}_{n_{k_{s}}}^x\}$ converges to $F^x$ at each continuity point of $F^x$. By the continuity of $F^x$, we have $\lim _{s\rightarrow \infty }\Vert \widehat{F}_{n_{k_{s}}}^x-F^x\Vert _{\infty }= 0$. This demonstrates that every subsequence of $\{\widehat{F}_{n_{k}}^x\}$ has a further subsequence converging uniformly to $F^x$. Therefore, $\lim _{k\rightarrow \infty }\Vert \widehat{F}_{n_{k}}^x-F^x\Vert _{\infty }= 0$ is derived. Note the uniform convergence holds for every $\omega \in \Omega _1$. It follows that $\Vert \widehat{F}_{n_{k}}^X-F^X\Vert _{\infty }$ converges almost surely to 0. Consequently, for every subsequence of $\Vert \widehat{F}_{n}^X-F^X\Vert _{\infty }$, there is a further subsequence that converges almost surely to 0. Therefore, $\Vert \widehat{F}_{n}^X-F^X\Vert _{\infty } {\mathop {\rightarrow }\limits ^\mathcal{P}} 0$ is demonstrated. $\square$

### Proof of Theorem 2

#### Proof

Following the notation given in the proof of Theorem 1, we consider the subsequence $\{\widehat{F}_{n_{k}}^x\}$ and

$$\Omega _1=\left\{ \omega \in \Omega : \lim _{k\rightarrow \infty }\Vert \widehat{H}^X_{n_{k}}- H^X\Vert _{\infty }=0 \text { and }\lim _{k\rightarrow \infty }\Vert \widehat{H}^X_{1n_{k}}- H^X_1\Vert _{\infty }=0\right\} .$$

We first work on a fixed $\omega \in \Omega _1$. Let $x=X(\omega ).$ We have shown in the proof of Theorem 1 that

$$\begin{aligned} \lim _{k\rightarrow \infty }\Vert \widehat{F}_{n_{k}}^x-F^x\Vert _{\infty }= 0, \; \lim _{k\rightarrow \infty }\Vert \widehat{H}^x_{0n_{k}}- H^x_0\Vert _{\infty }=0, \text { and } \lim _{k\rightarrow \infty }\Vert \widehat{H}^x_{1n_{k}}- H^x_1\Vert _{\infty }=0. \end{aligned}$$

Let $\mathbb {H}_{1,nm}^x$ denote the empirical subdistribution function of the $\widehat{Y}^x_{nj}$’s with $\widehat{\delta }^x_{nj}=1$ and let $\mathbb {H}_{0,nm}^x$ denote the empirical subdistribution function of the $\widehat{Y}^x_{nj}$’s with $\widehat{\delta }^x_{nj}=0$. The empirical log-likelihood divided by *m* is given by

$$\begin{aligned} \begin{aligned} l_{nm}(F)&=\frac{1}{m}\sum _{j=1}^{m}\left\{ \widehat{\delta }^x_{nj}\log F(\widehat{Y}^x_{nj})+(1-\widehat{\delta }^x_{nj})\log (1-F(\widehat{Y}^x_{nj}))\right\} \\&=\int \log Fd\mathbb {H}_{1,nm}^x+\int \log (1-F) d\mathbb {H}_{0,nm}^x, \end{aligned} \end{aligned}$$

where the term that does not depend on the distribution function *F* is omitted. From Groeneboom and Wellner (1992), $\widehat{F}_{nm}^x$ is the maximizer of $l_{nm}(F)$ over the class of all the distributions. Therefore, $\lim _{\varepsilon \rightarrow 0^{+}}\varepsilon ^{-1}\{l_{nm}((1-\varepsilon )\widehat{F}^x_{nm}+\varepsilon \widehat{F}^x_n)-l_{nm}(\widehat{F}^x_{nm})\}\le 0$, for which

A.6 $$\begin{aligned} \begin{aligned} \int \frac{\widehat{F}^x_n(u) }{\widehat{F}^x_{nm}(u)} d\mathbb {H}_{1,nm}^x(u)+ \int \frac{1- \widehat{F}^x_n(u)}{1-\widehat{F}^x_{nm}(u)} d\mathbb {H}_{0,nm}^x(u)\le 1. \end{aligned} \end{aligned}$$

Let *m* vary as *n* goes to infinity and we write $m=m_n$ to emphasize the dependence on *n*. For each $n_k$, we denote the index $n_km_{n_{k}}$ by $nm_{k}$ when there is no confusion. We consider the subsequence $\{\widehat{F}^x_{nm_k}\}$ corresponding to $\{\widehat{F}^x_{n_k}\}$. For fixed $\omega \in \Omega _1$, the randomness of $\mathbb {H}_{1,nm_k}^x$ is only from the samples $\eta _1,\dots ,\eta _{m_{n_k}}.$ Since the sequence $\{\Vert \mathbb {H}_{1,nm_k}^x-H^x_1\Vert _{\infty }\}$ is uniformly bounded, it follows by Prohorov’s theorem (van der Vaart 1998, Theorem 2.4) that there is a subsequence $\{\Vert \mathbb {H}_{1,nm_{k_{l}}}^x-H^x_1\Vert _{\infty }\}$ converging weakly to a random variable *Z*, where

$$\begin{aligned} \begin{aligned} \mathbb {E}Z^2&=\lim _{l\rightarrow \infty }\mathbb {E}\big \{\Vert \mathbb {H}_{1,nm_{k_{l}}}^x-H^x_1\Vert _{\infty }^2\big \} \\&\le 2\lim _{l\rightarrow \infty }\mathbb {E}\big \{ \Vert \mathbb {H}_{1,nm_{k_{l}}}^x-\widehat{H}_{1n_{k_l}}^x\Vert _{\infty } +\Vert \widehat{H}_{1n_{k_l}}^x-H^x_1\Vert _{\infty }\big \}= 0. \end{aligned} \end{aligned}$$

The subsequence $\{\Vert \mathbb {H}_{1,nm_{k_{l}}}^x-H^x_1\Vert _{\infty }\}$ converges in probability to 0. Therefore, there is a subsequence that converges almost surely to 0. Similarly, there is a subsequence of $\{\Vert \mathbb {H}_{0,nm_{k_{l}}}^x-H^x_0\Vert _{\infty }\}$ that converges almost surely to 0. Denote the subsequences by $\{\Vert \mathbb {H}_{1,nm_{r}}^x-H^x_1\Vert _{\infty }\}$ and $\{\Vert \mathbb {H}_{0,nm_{r}}^x-H^x_0\Vert _{\infty }\}$ with $\{n_k\}\subset \{n_{k_{l}}\}$. We define

$$\begin{aligned} \Lambda _1(\omega )=\left\{ \lambda \in \Lambda : \lim _{r\rightarrow \infty }\Vert \mathbb {H}_{1,nm_{r}}^x-H^x_1\Vert _{\infty }=0 \text { and } \lim _{r\rightarrow \infty }\Vert \mathbb {H}_{0,nm_{r}}^x-H^x_0\Vert _{\infty }=0\right\} . \end{aligned}$$

Then, $$\nu (\Lambda _1(\omega ))=1.$$

Next, we work on a fixed $\lambda \in \Lambda _1(\omega )$ and consider the subsequence $\{\widehat{F}^x_{nm_r}\}$. For $\delta \in (0,{1}/{2})$, we select *a*, *b* such that $F^x(a)=\delta$ and $F^x(b)=1-\delta .$ From (A.6), we have

$$\int _{(0,a]} \frac{\widehat{F}^x_{n_{r}}(u) }{\widehat{F}^x_{nm_r}(a)} d\mathbb {H}_{1,nm_r}^x(u) \le \int _{(0,a]} \frac{\widehat{F}^x_{n_{r}}(u) }{\widehat{F}^x_{nm_r}(u)} d\mathbb {H}_{1,nm_r}^x(u)\le 1.$$

It follows $\widehat{F}^x_{nm_r}(a)\ge \int _{(0,a]} \widehat{F}^x_{n_r}(u)d\mathbb {H}_{1,nm_r}^x(u)$, where the right side converges to $\int _{(0,a]} \widehat{F}^xdH_{1}^x$. Consequently, $1/\widehat{F}^x_{nm_r}(u)$ is bounded in [*a*, *b*] when *r* is large. Similarly, we derive $1/(1-\widehat{F}^x_{nm_r}(u))$ is bounded in [*a*, *b*] when *r* is large. By Helly’s lemma (van der Vaart 1998, Lemma 2.5), the sequence $\{\widehat{F}^x_{nm_r}\}$ has a subsequence $\{\widehat{F}^x_{nm_{r_{s}}}\}$ converging to a nondecreasing right continuous function $F^x_{2}$ at each continuity point of $F^x_{2}$. Following a similar method given in Lemma 4.1 of Groeneboom and Wellner (1992), we derive

$$\begin{aligned} \begin{aligned} \lim _{s\rightarrow \infty } \int _{[a,b]} \frac{\widehat{F}^x_{n_{r_{s}}} }{\widehat{F}^x_{nm_{r_{s}}}} d\mathbb {H}_{1,nm_{r_{s}}}^x+ \int _{[a,b]} \frac{1- \widehat{F}^x_{n_{r_{s}}} }{1-\widehat{F}^x_{nm_{r_{s}}}} d\mathbb {H}_{0,nm_{r_{s}}}^x=\int _{[a,b]} \frac{ F^x }{ F^x_{2}} d H_{1}^x+ \int _{[a,b]} \frac{1-F^x}{1- F^x_{2}} dH_{0}^x. \end{aligned} \end{aligned}$$

It follows from (A.6) and the monotone convergence theorem that

$$\begin{aligned} \begin{aligned} \int \frac{ F^x }{ F^x_{2}} d H_{1}^x+ \int \frac{1-F^x}{1- F^x_{2}} dH_{0}^x\le 1. \end{aligned} \end{aligned}$$

Note that

$$\int \frac{ F^x }{ F^x_{2}} d H_{1}^x+ \int \frac{1-F^x}{1- F^x_{2}} dH_{0}^x = \int \frac{ (F^x)^2 }{ 1-F^x_{2}} + \frac{(1-F^x)^2}{1- F^x_{2}} dH^x\ge 1.$$

Since the above equality only holds when $F_2^x=F^x$ and $F^x$ is continuous, we show $\lim _{s\rightarrow \infty }\Vert \widehat{F}^x_{nm_{r_{s}}}-F^x\Vert _{\infty }= 0$. This further demonstrates $\lim _{r\rightarrow \infty }\Vert \widehat{F}_{nm_{r}}^x-F^x\Vert _{\infty }= 0$. Define

$$\begin{aligned} W=\{(\omega ,\lambda )\in \Omega \times \Lambda : \omega \in \Omega _1, \lambda \in \Lambda _1(\omega )\}. \end{aligned}$$

From Fubini’s theorem, $\mu \times \nu (W)=1$. As the uniform convergence holds for every $(\omega ,\lambda )\in W$, $\Vert \widehat{F}_{nm_{r}}^X-F^X\Vert _{\infty }$ converges almost surely to 0. Then, for every subsequence of $\Vert \widehat{F}_{nm}^X-F^X\Vert _{\infty }$, there is a further subsequence that converges almost surely to 0. Therefore, we demonstrate $\Vert \widehat{F}_{nm}^X-F^X\Vert _{\infty } {\mathop {\rightarrow }\limits ^\mathcal{P}} 0$. $\square$
